# Thermal acclimation and metabolic scaling of a groundwater asellid in the climate change scenario

**DOI:** 10.1038/s41598-022-20891-4

**Published:** 2022-10-26

**Authors:** Tiziana Di Lorenzo, Ana Sofia P. S. Reboleira

**Affiliations:** 1 Research Institute on Terrestrial Ecosystems of the National Research Council, Via Madonna del Piano 10, 50019 Sesto Fiorentino, Firenze Italy; 2 Emil Racovita Institute of Speleology, Romanian Academy, Clinicilor 5, 400006 Cluj Napoca, Romania; 3 Departamento de Biologia Animal, Faculdade de Ciências, Centre for Ecology, Evolution and Environmental Changes (cE3c) & CHANGE – Global Change and Sustainability Institute, Universidade de Lisboa, Campo Grande, 1749-016 Lisbon, Portugal; 4 Natural History Museum of Denmark, University of Copenhagen, 2100 Copenhagen, Denmark

**Keywords:** Climate-change ecology, Freshwater ecology

## Abstract

Metabolic rate has long been used in animal adaptation and performance studies, and individual oxygen consumption is used as proxy of metabolic rate. Stygofauna are organisms adapted to groundwater with presumably lower metabolic rates than their surface relatives. How stygofauna will cope with global temperature increase remains unpredictable. We studied the thermal acclimation and metabolic scaling with body mass of a stygobitic crustacean, *Proasellus lusitanicus*, in the climate change scenario. We measured oxygen consumption rates in a thermal ramp-up experiment over four assay temperatures and tested two hypotheses: (i) *P. lusitanicus* exhibits narrow thermal plasticity, inadequate for coping with a fast-increasing thermal regime; and (ii) oxygen consumption rates scale with the body mass by a factor close to 0.75, as commonly observed in other animals. Our results show that *P. lusitanicus* has low thermal plasticity in a fast-increasing thermal regime. Our data also suggest that oxygen consumption rates of this species do not follow mass-dependent scaling, potentially representing a new trait of metabolic optimization in groundwater habitats, which are often limited in food and oxygen. Species with limited dispersal capacities and rigid metabolic guilds face extinction risk due to climate change and omitting groundwater ecosystems from climate change agendas emphasizes the unprotected status of stygofauna.

## Introduction

Mean surface temperature increments due to climate change are incontrovertible^1^. Current carbon emissions suggest that the societal aspiration to restrict global warming to about 2.0 °C above the pre-industrial period will hardly be achieved^2,3^. Groundwater is generally characterized by thermal stability, with temperatures highly correlating with mean annual temperatures on the surface^4^. Therefore, a significant large-scale increase in groundwater temperatures is expected in the next years^5^. Since the 1980s, groundwater temperature increasing in the range of 0.7 to 3.0 °C has been observed in some unconsolidated alluvial aquifers^6^ and groundwaters of densely urbanized areas^7^. Temperatures of shallow groundwaters worldwide are projected to rise by 3–5 °C within the next century^8,9^. Although consequences of global climate change on aquatic biodiversity are widely predictable^10^, groundwater biodiversity is often disregarded^11^.

Groundwater is a highly diverse ecosystem, hosting over 7000 known animal species currently extinct on the surface (mostly arthropods^12^) and 2–6 × 10^26^ cells of microorganisms (prokaryotes^13^; fungi and a viral repository^14^), compressed into just ~ 19% of the world’s area^15^. Groundwater-adapted biodiversity is mainly composed of short-range species with restricted distributions [e.g.^16,17^]. Many are representatives of old lineages currently extinct at the surface, with high phylogenetic and conservationist value^18^. Obligate-groundwater species (hereafter referred as to “stygobites”) are adapted to environments with no light, limited food resources, low oxygen and low thermal amplitude^19^. Thermal acclimation is a phenotypic response to environmental temperature variation^20,21^ that has important implications for coping with global climate change^22^. Ectotherms from areas with high variability in temperature have greater acclimation abilities than those residing in habitats with low variability^20^. Therefore, stygobitic ectotherms are expected to exhibit a low thermal acclimation ability^23–29^. Recent studies suggest that some stygobitic species might have lost thermal acclimation mechanisms to adapt to groundwater^21^. However, our current knowledge on thermal acclimation of stygobitic species remains limited^24,30,31^.

Standard oxygen consumption rate (i.e., the oxygen required, in a time unit, for minimal resting lifestyle) is the most used variable in studies on animal short-term (hours or day) thermal acclimation^32–34^. When the environmental temperature rises, standard oxygen consumption rates of ectotherms also vary as a response to a sudden shift in temperature^35^. Standard oxygen consumption rates also change with the size of the organisms and this size-related effect is called scaling^35,36^. This relationship is described by a linear model where the scaling factor (also known as “mass exponent”) is in the range of 0.66 to 0.75 for most animal taxa^35–37^. Studies concerning thermal acclimation of stygobitic species are scarce^27,38^, only two studies investigated the scaling of standard oxygen consumption rates with body mass in stygobitic crustaceans^28,39^.

In this study, we measured the standard oxygen consumption rates of *Proasellus lusitanicus* (Frade, 1938), a stygobitic asellid (Crustacea Isopoda: Asellidae) endemic to a single karst aquifer in Portugal. Standard oxygen consumption rates were measured in a thermal ramp-up experiment from the groundwater temperature of the collection site to the one expected in the next 90 years in the aquifers of the 45° parallel North^8^. We also measured the scaling of oxygen consumption rates with body mass for this species. First, we hypothesized that *P. lusitanicus* would exhibit narrow thermal plasticity, inadequate for coping with a rapid-increasing thermal regime. Secondly, we assumed that the oxygen consumption rates of *P. lusitanicus* would scale with body mass showing a scaling exponent in the range of 0.66 to 0.75, as commonly measured for other animals. Findings are contextualized with previous studies.

## Materials and methods

### Collection and acclimation to laboratory conditions

**Figure 1:**
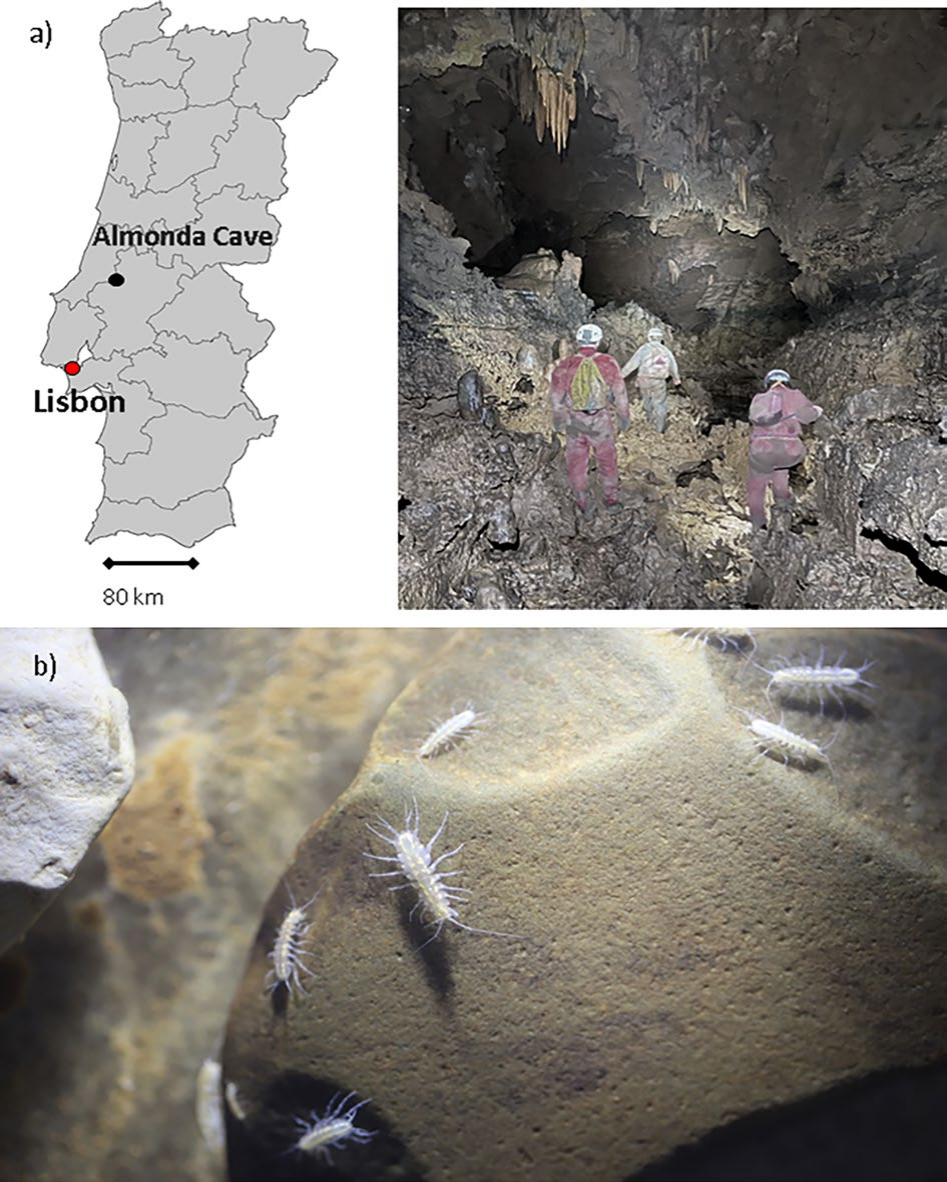
Sampling site of *Proasellus lusitanicus* (Frade, 1938) in this study. (**a**) Location of Almonda Cave, Estremenho karst massif, Portugal, and researchers at the collection site. (**b**) Adult individuals in the cave.

The stygobitic asellid *Proasellus lusitanicus* is an endemism living in the caves of Estremenho karst massif in Central Portugal (Fig. 1a), the largest karst aquifer of the Iberian Peninsula. The aquifer was the primary freshwater source for Lisbon in the last century^40^, and previous analyses indicated that groundwater from Estremenho karst massif was uncontaminated^41^, precisely, PAHs, PCBs, pesticides and heavy metals concentrations were below European detection limits^41^. *Proasellus lusitanicus* shows some of the most typical adaptive traits to groundwater lacks eyes and pigmentation, has appendage hypertrophy and breathes through branchiae (Fig. 1b)^41–43^. On September 2nd 2021, we collected only adults in a small stream running towards the ‘Christmas siphon’ of Almonda Cave (39° 30′ 20.05′′ N, 8° 36′ 56.63′′ W; Fig. 1a) using a plastic pipette. We put the individuals in a plastic container filled with groundwater and sediments from the collection site. We placed the container in a cooler and transported the individuals to the laboratory within a few hours from collection, as indicated in Castaño-Sánchez et al.^43^. We measured electrical conductivity (508 μS/cm), oxygen (6.9 mg/L), pH (7.1) and temperature (17 °C) in the field at the collection site using a portable multiparametric probe (WTW MULTI 3430).

**Figure 2:**
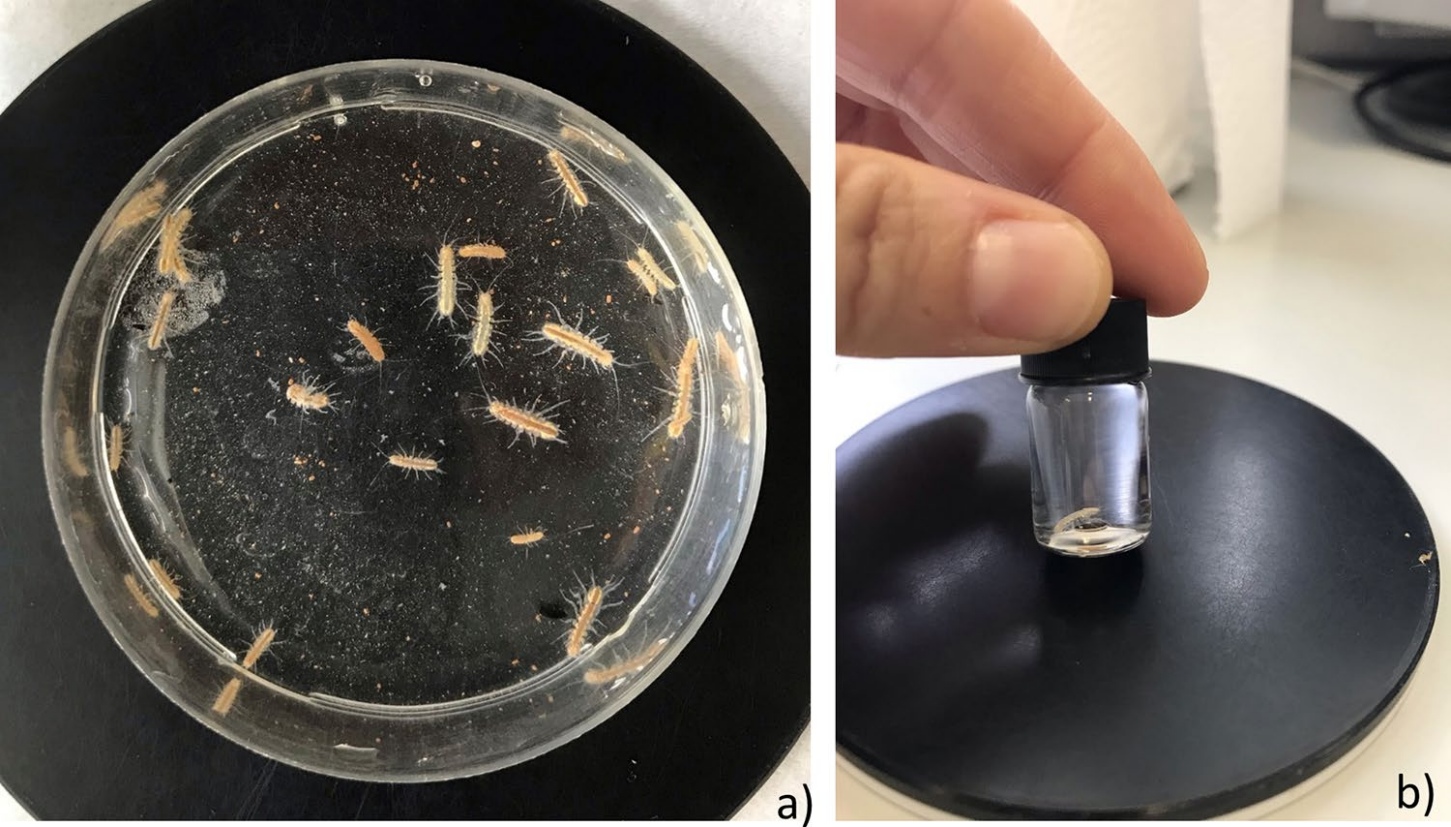
The stygobitic *Proasellus lusitanicus.* (**a**) Adult individuals; (**b**) resting position of the individuals in the measurement chambers.

In the laboratory, we selected up 20 adult individuals of different sizes (Fig. 2a). We kept them in a 1-L aquarium, in constant darkness, at the temperature of the collection site (17 °C ± 0.2 °C) in a thermo-regulated cabinet for 2 weeks to let them acclimate to laboratory conditions. Since guidelines suggest providing at least 1 mL of groundwater per individual^44^, we filled the aquarium with 800 mL of groundwater from the collection site. The dissolved oxygen concentration was measured three times during acclimation and was in the range of 6.5 to 7.0 mg/L. We did not change the groundwater in the aquarium during the two weeks to avoid providing additional stress to the animals^44^. According to previous studies^44^ and our personal observations, *P. lusitanicus* is a deposit feeder. For this reason, we provided the collected individuals with homogenized cave sediments by using a micro-spoon spatula (one scoop per individual). Five pebbles (5 cm in diameter) from the cave stream were provided as shelters. We offered no additional artificial food.

### Thermal ramp-up experiment

**Figure 3:**
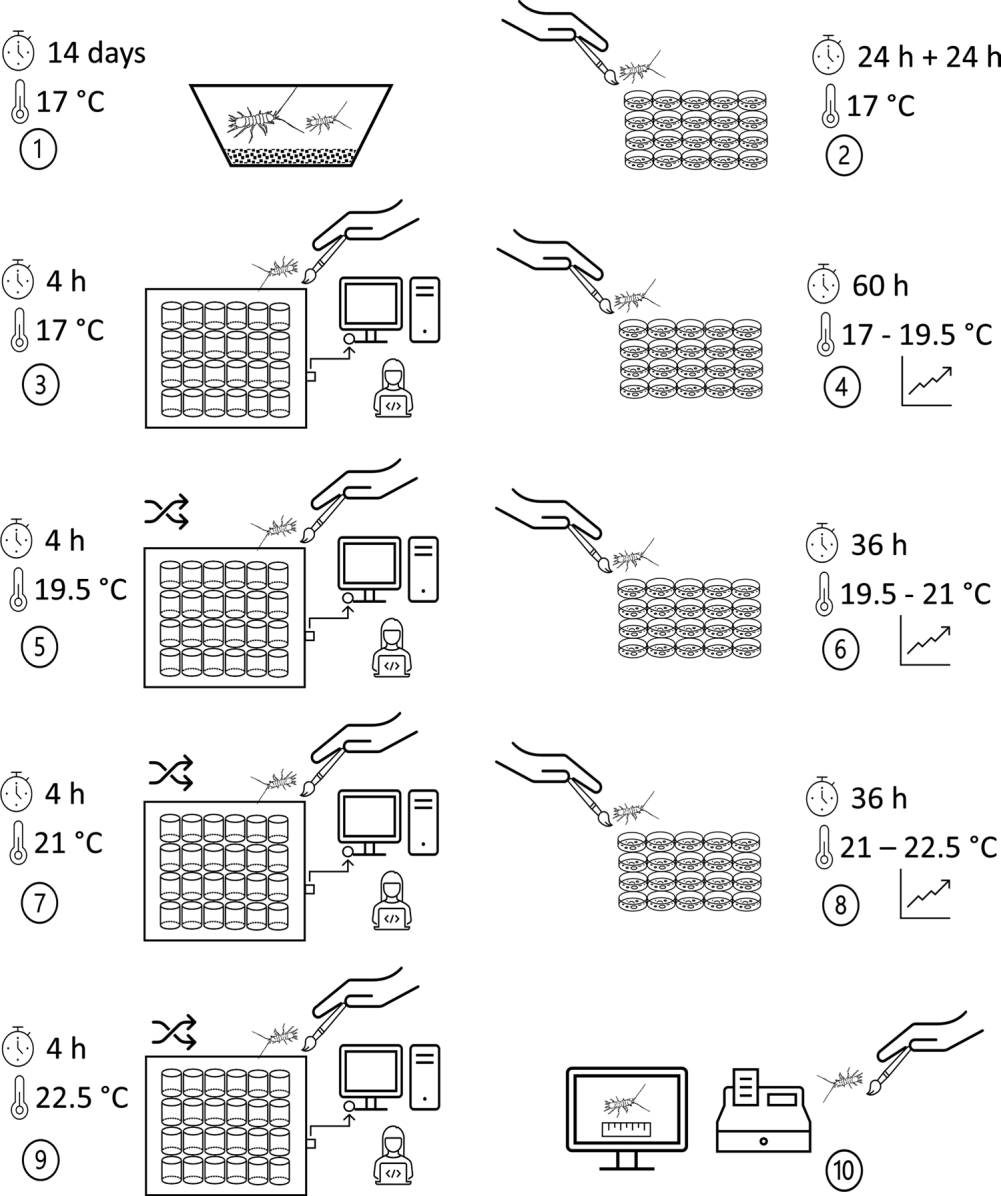
Phases of the thermal ramp-up experiment performed with twenty adult individuals of *Proasellus lusitanicus*. Circled numbers indicate phases. Phase I acclimation to laboratory conditions; Phase II acclimation to new medium and vials; Phase III measurements; Phase IV acclimation to a new temperature; Phase V measurements; Phases VI, VII, and VIII acclimation to a new temperature and measurements; and Phase X dry mass estimates.

We evaluated the thermal acclimation of *P. lusitanicus* by measuring standard oxygen consumption rates in a ramp-up experiment using four assay temperatures (17, 19.5, 21, 22.5 °C). Experimental phases are shown in Fig. 3. Phase I (acclimation to laboratory conditions; Fig. 3): this phase was described in the previous paragraph. Phase II (acclimation to new medium and vials; Fig. 3): after 14 days of acclimation to laboratory conditions, we gently picked up one individual at a time by using a soft brush and transferred them into 2-mL glass vials (one individual per vial). We filled each vial with 1 mL of commercial water (electrical conductivity: 110 µS/cm; pH: 6.40; SiO_2_^−^: 58 mg/L; Ca^2+^: 14 mg/L; Na^+^: 27 mg/L; Cl^−^: 20 mg/L; HCO_3_^−^: 469 mg/L) and 1 mL of groundwater from the collection site. We also added one scoop of homogenized cave sediments (dosed by using a micro-spoon spatula). Individuals were kept at 17 °C for acclimation to the new medium and vials for 24 h, according to Di Lorenzo et al.^44^. After 24 h, we replaced the medium with 2 mL of commercial water and removed the sediment to ensure that the digestive tracts of each individual would be empty during subsequent oxygen consumption measurements. We removed feces to avoid oxygen overshoot due to animal wastes. The specimens were kept like this further 24 h. Phase III (measurements; Fig. 3): before starting the measurements, we transferred the individuals into the measurement chambers. The chambers were 2-mL vials equipped with planar, pre-calibrated oxygen sensor spots (4 mm diameter) with optical isolation glued onto the bottom (Loligo Systems, Viborg, Denmark). We filled each chamber with 2 mL of commercial water. The chambers were integrated with a reader consisting of a 24-channel fluorescence-based respirometry system (SDR Sensor Dish Reader; PreSens Precision Sensing GmbH, Regensburg, Germany). We connected the reader to a computer to measure the oxygen consumption rates of 20 individuals of *P. lusitanicus* and four blank controls (i.e., vials without animals) simultaneously (Fig. 3). We placed the reader inside the thermostatic cabinet at the appropriate testing temperatures 18 h before the beginning of the measurements to equilibrate the equipment temperature. Water temperature in each chamber was recorded with a temperature logger already integrated into the device. After animal loading, the chambers were sealed by screw caps and inspected for air bubbles before being placed on the reader and into the thermostatic cabinet at 17 °C. We started the measurements after 2 h from animal loading to let temperature equilibrate^29^. Oxygen concentration in each chamber was recorded every 5 min for 4 h after temperature re-equilibration. Linear decrease of oxygen concentration was used to calculate oxygen consumption rates in ng O_2_/h for each individual. We corrected oxygen consumption rates for microbial respiration measured in the control chambers. Phase IV) (acclimation to a new temperature; Fig. 3, time proportional to the thermal increment): at the end of the measurements at 17 °C, we returned the animals to the 2-mL glass vials filled with commercial water. We provided one scoop of homogenized cave sediments in each vial. We kept the vials in the thermostatic cabinet and increased the temperature by 0.041 °C/h. We deemed this ramping rate more appropriate for stygobitic species than the faster ramping rates of 1 °C/min or 1 °C/h used for other ectotherms [e.g.^45^]. When the next assay temperature was reached, we cleaned the sediment and feces from the vials and let the animals acclimate to the new temperature for 24 h before measurements. Phase V (measurements; Fig. 3): oxygen consumptions were measured as described in Phase III. The procedures described in phases IV and V (acclimation to a new temperature and measurements) were repeated to perform the measurements at 21 °C (Phases VI and VII) and 22.5 °C (Phases VIII and IX). We always used the same individuals, randomly switching the measurement chambers (and the relative oxygen sensors) to assure the independence of the measurements at each assay temperature. All measurements were run between 11 a.m. and 7 p.m. time period. Phase X) (dry mass estimates; Fig. 3): at the end of the experiment, the animals were individually photographed and then measured using ImageJ software^46^. Afterwards, wet mass was measured on a laboratory scale (A&D, model ER-120A) with 0.1 mg accuracy. Finally, dry mass was estimated by weighting the individuals after placing them between two sheets of blotting paper to remove water.

The activity of adult individuals of *P. lusitanicus* in the measurement chambers was inspected in preliminary experiments with half-hourly interval observations during 4 h. In these preliminary trials, the individuals predominantly sat still on the top of the oxygen sensors (Fig. 2b), as observed for some cave amphipod species^39^. Therefore, the measured rates of oxygen decline were the best estimate of standard oxygen consumption rates, according to Clarke and Fraser^33^. Oxygen consumption rates of the individuals which died at temperatures > 17 °C (1 dead individual at 19.5 °C, 2 at 21 °C and 2 at 22.5 °C) and a female that became ovigerous during thermal acclimation at 21 °C, were discarded from the subsequent analyses.

We used a one-way permutational analysis of variance (PERMANOVA^47^) followed by a permutational pairwise *t*-test to highlight potential differences in oxygen consumption rates at the four temperatures. We used a resemblance matrix based on Euclidean distances of raw data. We applied a one-way design consisting of one fixed factor (temperature) with four levels. We examined the model under the Type I partitioning of sum of squares. We also used permutation of residuals under a reduced model as it yields the best power and the most accurate type I error for matrices with a few data^47^. We used PERMANOVA because it does not rely on ANOVA assumptions relating to the data distribution. Nevertheless, we performed a Levene’s test prior to PERMANOVAs to account for the potential heterogeneity of the variances among the four levels of the fixed factor^47^. The significance level (α) was set at 0.05 since permutational tests do not require alpha correction for multiple groups^47^. All analyses were performed with E-PRIMER and PERMANOVA + software v. 6^47^.

### Metabolic scaling

Oxygen consumption rates and body mass are related as in Eq. (1)^48^:

1 $$OCR=\mathrm{a}{M}^{b},$$

where *OCR* is oxygen consumption rate, *M* is dry mass, *a* is a proportionality constant and *b* is the scaling factor, which falls within the range of 0.66–0.75 for most animals [e.g.^49^]. Equation (1) can be conveniently log-transformed as in Eq. (2):

2 $$log\left(OCR\right)=\log\left(a\right)+b \times \log\left(M\right).$$

To assess the linear scaling of log oxygen consumption rates of *P. lusitanicus* with log body mass, we fitted a permutational Ordinary Least Square (OLS) regression model to the data collected at 17 °C (groundwater temperature at the collection site). We also fitted OLS models to data at the three assay temperatures > 17 °C to assess potential scaling variation due to thermal stress. We repeated the analyses after removing potential outliers from data at 19.5, 21 and 22.5 °C, where outliers were values ≤ 0.01 ng O_2_/mg h (Supplementary Table S1). Permutation tests are preferred when data distribution departs from normality^50^, as was the case for the present data even after the exclusion of the above mentioned extreme values. The significance (α) of the intercept and slope was set at 0.05, number of permutations of independent variable was 999, while the fit of the models was assessed by using R^2^. The analyses and plots were performed by using R vs. 4.1.1 and the library “ape”^51^.

### Respiration rates of aquatic isopod species

To contextualize the rates of oxygen consumption of *P. lusitanicus* with previous studies, we surveyed the literature for respiration rates of asellids by performing a search in Clarivate Analytics Web of Science. We used the keywords as follows: TS = "isopod*” AND TS = (“oxygen” OR “consumption” OR “SRR” OR “respiration”) AND TS = (“aquatic” OR “freshwater” OR “groundwater”), where TS denotes a search for ‘Topic’. The asterisk is used to match all words beginning with that string of characters. We used the PlotDigitizer 2.6.8 software (http://plotdigitizer.sourceforge.net/) to extract data available only in graphs. Data retrieved from the literature were converted to ng O_2_/mg h for comparative purposes whenever necessary. No temperature corrections were made, and temperatures at which each study was conducted were annotated.

## Results

Dry mass of the adult individuals of *Proasellus lusitanicus* was in the range of 1.7–9.5 mg (mean ± SD: 4.7 ± 3.3 mg). Body length was 4.2–7.2 mm (mean ± SD: 5.2 ± 1.3 mm). Oxygen consumption rates of adult individuals were in the range of 21.3–259.5 ng O_2_/mg h at 17 °C, 0.04–191.4 ng O_2_/mg h at 19.5 °C, 0.03–142.4 ng O_2_/mg h at 21 °C and 0.01–91.9 ng O_2_/mg h at 22.5 °C (Supplementary Table S1). Mean values at the four assay temperatures are reported in Table 1. At temperatures > 17 °C, 8 individuals (with body mass in the range of 2.2–8.8 mg) had extremely low oxygen consumption rates. However, they remained alive until the end of the experiment (Supplementary Table S1). Five individuals died at temperatures > 17 °C. The main PERMANOVA test highlighted that oxygen consumption rates differed at the four assay temperatures (PERMANOVA: Pseudo-F_3,55_ = 4.45, p = 0.0048, perms = 9953). However, the differences were significant between 17.0 and 22.5 °C (pairwise t-test: t = 3.28, p = 0.0004, perms = 8417), 19.5 and 22.5 °C (pairwise t-test: t = 3.27, p = 0.0024, perms = 7990) and 21.0 and 22.5 °C (pairwise t-test: t = 2.52, p = 0.0179, perms = 7714).

**Table 1:** Mean (μ), standard deviation (SD), minimum (Min) and maximum (Max) values of oxygen consumption rates (ng O_2_/mg h) of the individuals of *Proasellus lusitanicus* used in this study at four assay temperatures.

T (°C)	μ	SD	Min	Max
17.0	85.82	70.23	21.35	259.50
19.5	70.26	51.01	0.04	191.38
21.0	53.67	42.40	0.03	142.38
22.5	19.77	26.95	0.01	91.85

**Figure 4:**
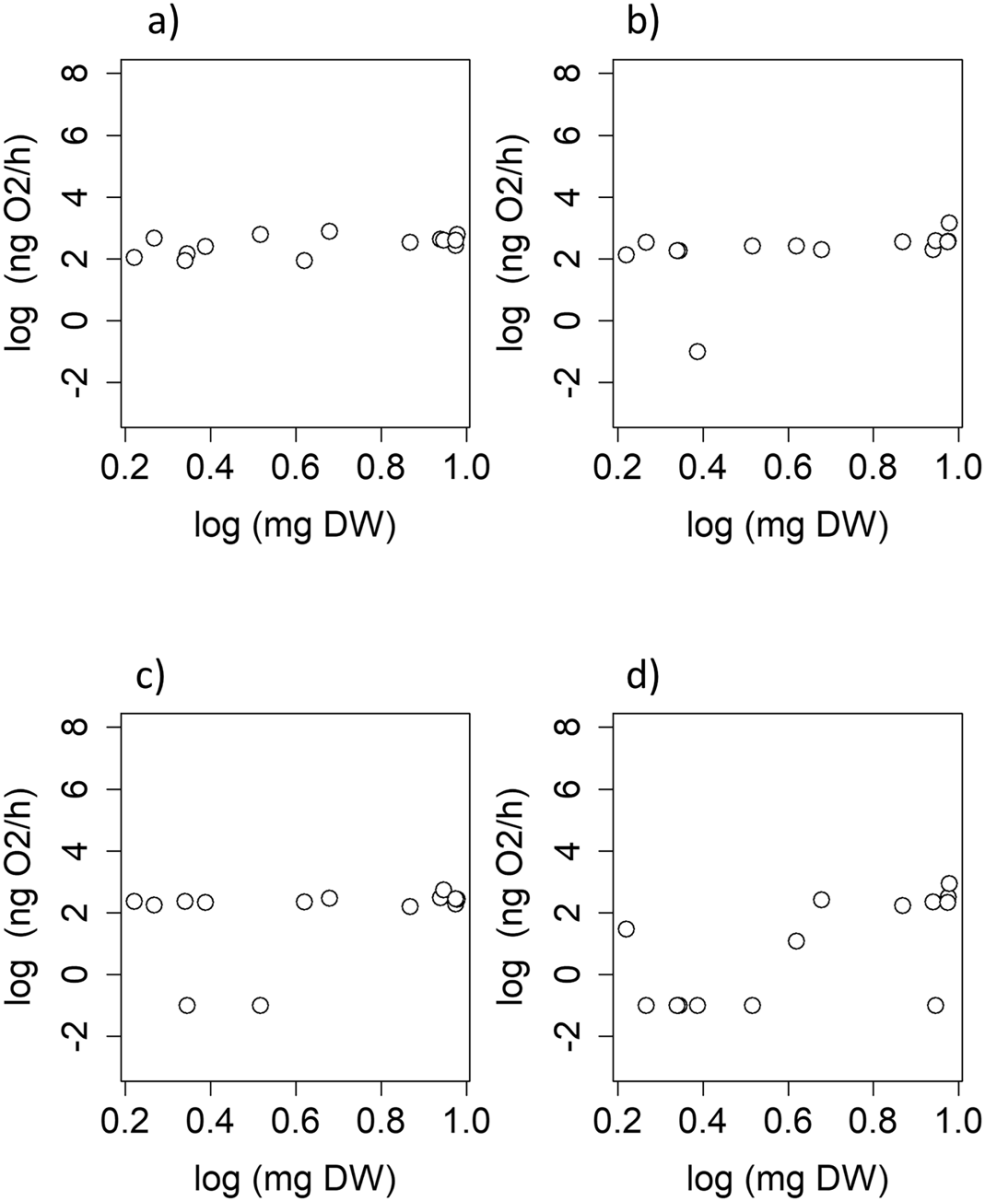
Log–log scatter plots of oxygen consumption rates vs. body mass of adults of *Proasellus lusitanicus* at: (**a**) 17 °C; (**b**) 19.5 °C; (**c**) 21 °C; (**d**) 22.5 °C. Model parameters are reported in Table 2.

The permutational OLS regression highlighted that log oxygen consumption rates of *P. lusitanicus* at 17 °C were not linearly correlated to log body mass (Table 2, Fig. 4). We obtained the same result across all assay temperatures. After outliers’ removal, the OLS model was significant at 19.5 °C, showing an intercept of 2.14 ng O_2_/h and a slope of 0.49 ng O_2_/h (Table 2, Fig. 4). However, the model explained 34% of data variability (Table 2).

**Table 2:** Summary of the permutational ordinary least square regression models used to assess the linear scaling of log oxygen consumption rates with log body mass in *Proasellus lusitanicus* (Frade, 1938) at the four assay temperatures.

T (°C)	Parameter	Coefficient	Standard error	*t*-statistic	p-value	R^2^
		ng O_2_/h	ng O_2_/h			
17	Intercept	2.12	0.19	11.07	** < 0.001**	0.24
	Slope	0.53	0.27	1.96	0.073	
19.5	Intercept	1.41	0.60	2.33	**0.038**	0.14
	Slope	1.24	0.86	1.45	0.172	
21	Intercept	0.98	0.81	1.22	0.243	0.12
	Slope	1.43	1.13	1.27	0.228	
22.5	Intercept	− 1.58	0.87	− 1.79	0.097	0.42
	Slope	3.71	1.24	2.97	**0.012**	
**After outliers’ removal**						
17	Intercept	2.12	0.19	11.07	** < 0.001**	0.24
	Slope	0.53	0.27	1.96	0.073	
19.5	Intercept	2.14	0.15	14.55	** < 0.001**	0.34
	Slope	0.49	0.20	2.37	**0.036**	
21	Intercept	2.27	0.10	22.40	** < 0.001**	0.14
	Slope	0.17	0.13	1.27	0.234	
22.5	Intercept	0.88	0.50	1.74	0.132	0.54
	Slope	1.65	0.61	2.67	**0.036**	

Significance was set at p-value < 0.05. Slope is in the range of 0.66–0.75 for most animals [e.g.^49^].

Significant values are in bold.

Oxygen consumption rates of other stygobitic isopod species (Table 3) varied from 20 ng O_2_/mg h (*P. lusitanicus* at 22.5 °C; this study) to 1750 ng O_2_/mg h (*P. valdensis* 1^27^). Oxygen consumption rates of *P. lusitanicus* at 19 °C and 22.5 °C (mean values: 70 and 20 ng O_2_/mg h, respectively) were much lower than those of *P. valdensis* 1 (1250 ng O_2_/mg h at 19 °C and 1150 ng O_2_/mg h at 22 °C^27^), *P. valdensis* 2 (1200 ng O_2_/mg h at 19 °C;^27^) and *P. valdensis* n. sp. 2 (1700 ng O_2_/mg h at 19 °C^27^).

**Table 3:** Oxygen consumption rates (OCR; ng O_2_/mg h) of stygobitic (SB) and non-stygobitic (nSB) isopod species at different temperatures (T).

Taxon	Location	OCR	T (°C)	Ref.
*Asellus aquaticus carniolicus* (nSB)	Cerkniško jezero (Slovenia)	25	10	^52^
*Asellus aquaticus* (nSB)	Rhône River (France)	196	11	^38^
*Asellus aquaticus cavernicolus* (SB)	Zelške jame (Slovenia)	25	10	^52^
*Stenasellus virei* (SB)	Groundwater of River Tarn (France)	64	11	^2^
*Proasellus valdensis* 1 (SB)	La Balme caves (France)	350	2	^27^
*Proasellus valdensis* 1 (SB)	La Balme caves (France)	350	4	^27^
*Proasellus valdensis* 1 (SB)	La Balme caves (France)	750	7	^27^
*Proasellus valdensis* 1 (SB)	La Balme caves (France)	850	10	^27^
*Proasellus valdensis* 1 (SB)	La Balme caves (France)	850	13	^27^
*Proasellus valdensis* 1 (SB)	La Balme caves (France)	1750	16	^27^
*Proasellus valdensis* 1 (SB)	La Balme caves (France)	1250	19	^27^
*Proasellus valdensis* 1 (SB)	La Balme caves (France)	1150	22	^3^
*Proasellus* n. sp.1 (SB)	Baume la Fraîte Cave (France)	400	2	^27^
*Proasellus* n. sp.1 (SB)	Baume la Fraîte Cave (France)	370	4	^27^
*Proasellus* n. sp.1 (SB)	Baume la Fraîte Cave (France)	750	7	^27^
*Proasellus* n. sp.1 (SB)	Baume la Fraîte Cave (France)	600	10	^27^
*Proasellus* n. sp.1 (SB)	Baume la Fraîte Cave (France)	1200	13	^27^
*Proasellus valdensis* 2 (SB)	Mouthe spring (France)	450	2	^27^
*Proasellus valdensis* 2 (SB)	Mouthe spring (France)	370	4	^27^
*Proasellus valdensis* 2 (SB)	Mouthe spring (France)	470	7	^27^
*Proasellus valdensis* 2 (SB)	Mouthe spring (France)	650	10	^27^
*Proasellus valdensis* 2 (SB)	Mouthe spring (France)	600	13	^27^
*Proasellus valdensis* 2 (SB)	Mouthe spring (France)	1100	16	^27^
*Proasellus valdensis* 2 (SB)	Mouthe spring (France)	1200	19	^27^
*Proasellus* n. sp.2 (SB)	Borne aux Cassots Cave (France)	400	2	^27^
*Proasellus* n. sp.2 (SB)	Borne aux Cassots Cave (France)	450	4	^27^
*Proasellus* n. sp.2 (SB)	Borne aux Cassots Cave (France)	470	7	^27^
*Proasellus* n. sp.2 (SB)	Borne aux Cassots Cave (France)	650	10	^27^
*Proasellus* n. sp.2 (SB)	Borne aux Cassots Cave (France)	900	13	^27^
*Proasellus* n. sp.2 (SB)	Borne aux Cassots Cave (France)	1350	16	^27^
*Proasellus* n. sp.2 (SB)	Borne aux Cassots Cave (France)	1700	19	^27^
*Proasellus lusitanicus* (SB)	Estremenho massif (Portugal)	86	17	^this study^
*Proasellus lusitanicus* (SB)	Estremenho massif (Portugal)	70	19.5	^this study^
*Proasellus lusitanicus* (SB)	Estremenho massif (Portugal)	54	21	^this study^
*Proasellus lusitanicus* (SB)	Estremenho massif (Portugal)	20	22.5	^this study^

## Discussion

The results of our study indicate that *Proasellus lusitanicus* has a low thermal acclimation ability. At 22.5 °C, oxygen consumption rates of adult individuals of this species were reduced by > 75% as compared to those at the temperature of the collection site (17 °C). In addition, some adults died at temperatures exceeding that of the collection site by ≥ 2.5 °C. Finally, our results indicate that eight adult individuals might have experienced heat rigor, i.e., rigor of living tissue caused by exposure to excessive but not immediately lethal temperatures^53^ at > 17 °C. We suspect that uropod movement was highly reduced in these individuals because their oxygen consumption rates were several orders of magnitude lower than those of other individuals engaged in the experiment. Our results suggest that *P. lusitanicus* is a stenothermic species, as observed for other stygobitic *Proasellus* species living in thermally buffered environments^27^. On the other hand, previous studies showed that *P. valdensis* (Chappuis, 1948), which lives in habitats with groundwater temperatures ranging from 4.1 to 11.8 °C (though each population resides in a thermally buffered habitat^27^), has a wider thermal tolerance than *P. lusitanicus*. These differences are consistent with theories suggesting that ectotherms living in areas with high variability in temperature acclimate better than those residing in habitats with low thermal variations^20^. Accordingly, *P. lusitanicus* exhibits a much-restricted distribution as compared to *P. valdensis*. The significant decrease in oxygen consumption rates at temperatures diverging from that at the collection site, also associated with some fatalities at temperatures > 17.0 °C in the case of *P. lusitanicus*, is suggestive of metabolic alteration during acclimation that should be further analyzed. Variability in metabolic end-products, such as lactate and succinate, and immune defenses are likely to be the causes^27^. Movement tracking and behavioral analyses could be useful for exploring how metabolic alterations at elevated temperatures reverberate, not only on single populations, but also on community and ecosystem services. For instance, some individuals of *P. valdensis* are known to reduce their locomotor activity by about 40% when temperature diverges from that of the collection site by ≥ 3 °C^27^. This behavioral alteration likely results in reduced efficiency in foraging and mating, and alterations in interactions associated with predation or competition. Our results suggest that if the worst global warming scenario is met in the next century, *P. lusitanicus* will likely go extinct. Population declines will be imputed to this species’ inability to acclimate fast enough to track increasing temperatures, as speculated for other species [e.g.^31,54,55^]. We speculate that there will not be possibility of recolonization from other geographic areas because of the restricted distribution of this endemic species^56,57^. This will inevitably have detrimental cascading effects involving essential groundwater ecosystem services^23,24,58,59^.

Oxygen consumption rates of *P. lusitanicus* fall within the range reported for other stygobitic isopod species and this supports the reliability of our measurements. However, *P. lusitanicus* has oxygen consumption rates lower than other *Proasellus* species. Such metabolic differences among closely phylogenetically-related species are not unlikely and are related to multiple factors. Previous studies have revealed that most of the variance in oxygen consumption rates of crustacean species can be attributed to temperature variability at the collection site and differences in body mass^60^. Thermally-stable environments require low energy for thermoregulation. Ammonia and phosphate excretion rates also play a role^60^. The epigean asellid species *Asellus aquaticus* has oxygen consumption rates twice the values of *P. lusitanicus*. Previous studies supported the theory that stygobitic species evolved a lower metabolism, compared to that of close-related surface species, as adaptations towards some environmental features of groundwater environments [e.g.^28,38,39,52^]. Reduced metabolism is advantageous for organisms living in habitats with chronically low and/or discontinuous food and oxygen supplies^39,61^. A metabolism independent on body mass may be seen a further advantage in groundwater habitats, as largest individuals may conserve energy more efficiently. Our results suggest that log oxygen consumption rates of *P. lusitanicus* do not depend linearly on log body mass (at least in the mass range of 1.7–9.5 mg) at the temperature of the collection site. We consistently observed the lack of linear scaling across the assay temperatures, except at 19.5 °C after outliers’ removal. Although the OLS model was significant at that temperature, its reliability is questionable because of the low percentage of explained variance. The apparent lack of linear scaling for *P. lusitanicus* is a result that diverges from most other animals^36,37^. Both endothermic and ectothermic animals have log oxygen consumption rates that scale linearly with log mass with a scaling factor *b* close to 0.75^49^, at least within the limited range of "biologically relevant" temperatures (0° and 40 °C^36^). However, the apparent lack of linear scaling is not an overall novelty for groundwater species. It has been already observed in two other stygobitic crustacean species. Log oxygen consumption rates of the stygobitic copepod *Diacyclops belgicus* Kiefer, 1936 seem not to scale linearly with body mass, at least in the mass range of 3.0–5.0 ng^28^. Similarly, log oxygen consumption rates of the stygobitic amphipod *Gammarus acherondytes* Hubricht & Mackins, 1940 do not scale with log body mass in the mass range of 1.5–9.3 mg^39^. Wilhelm et al.^39^ assumed that the lack of scaling could represent an adaptation to groundwater habitats where temporal unavailability of food and oxygen can be pronounced^62,63^. In particular, the independence of oxygen consumption rates from mass is an evident advantage for large individuals in food-deprived habitats. Other explanations had been called into question by Wilhelm et al.^39^ and then discarded. For instance, lack of scaling in *G. acherondytes* could have been due to differences in individual behavioral activity, with larger amphipods less active than the smaller ones. However, Wilhelm et al.^39^ observed that the individuals engaged in their experiment spent most of their time sitting on the oxygen sensors, showing no evident differences in movement or behavior. We can state the same for *P. lusitanicus*. A further explanation is relative to the mass range, which might have been too small to detect a statistically-significant relationship. Wilhelm et al.^39^ stated that they covered the range of mass (1.5–9.3 mg) for the collection site of *G. acherondytes*. We can say the same (1.7–9.5 mg) for the individuals of *P. lusitanicus* used in our experiment. However, data limitations in terms of both investigated species and measurements prevent claiming a novel metabolic guild limited to stygofauna, and further studies are necessary to explore this possibility. Furthermore, the apparent lack of linear scaling of log oxygen consumption rates with log mass only applies to adult individuals. Oxygen consumption rates of juvenile stages of *P. lusitanicus* and *G. acherondytes* have not been measured yet. Log oxygen consumption rates of juveniles of *D. belgicus* scale isometrically with log mass, i.e. with a scaling factor *b* close to 1^28^. However, isometric scaling during ontogeny is commonly observed in many species and interpreted as a response to rapid growth rates necessary to face high juvenile mortality and predation^64^.

## Conclusions

Our findings indicate that stenothermic species, such as the stygobitic *P. lusitanicus*, might be at extinction risk due to climate change, because of the reduced number of suitable habitats that may act as thermal refuges in next future and the inability to disperse to other habitats due to belowground isolation. Even if some adaptation is expected, the temporal scale at which key physiological traits (such as thermal plasticity) evolve is uncertain and the extinction risk of this stygobitic species is high^19^. Stygobitic crustaceans are key species for the groundwater ecosystem, and their extinction threatens the entire ecosystem services, which are vital for all life on Earth^19,23,59^. This study is particularly relevant to understand the thermal acclimation ability of stygobitic crustaceans, and by extension its implications in the ecological integrity of groundwater habitats^24^. Furthermore, the omission of groundwater ecosystems from climate change agendas^65,66^ marginalizes their ecological importance, leaving stygofauna substantially unprotected. Our findings also aim at stimulating open questions on metabolic adaptations of stygobitic species to thermally-stable, nutrient- and oxygen-poor, groundwater habitats.

## Supplementary Information

Supplementary Table S1.

## Data Availability

All data generated or analyzed during this study are included in this paper and Supplementary File.
