# Supplementary material for: Thermal acclimation and metabolic scaling of a groundwater asellid in the climate change scenario
Source: Sci Rep. 2022 Oct 26;12:17938. doi: 10.1038/s41598-022-20891-4 (PMC9605946; doi:10.1038/s41598-022-20891-4)
Supplement: Supplementary file 1 — Supplementary Table S1. [file 41598_2022_20891_MOESM1_ESM.docx]

Thermal acclimation and metabolic scaling of a groundwater asellid in the climate change scenario

Tiziana Di Lorenzo^1,2,3^, Ana Sofia P.S. Reboleira^3,4*^

^1^Research Institute on Terrestrial Ecosystems of the National Research Council, Via Madonna del Piano 10, 50019, Sesto Fiorentino, Firenze, Italy

^2^Emil Racovita Institute of Speleology, Romanian Academy, Clinicilor 5, Cluj Napoca 400006, Romania

^3^Centre for Ecology, Evolution and Environmental Changes (cE3c) & CHANGE – Global Change and Sustainability Institute, and Departamento de Biologia Animal, Faculdade de Ciências, Universidade de Lisboa, Campo Grande 1749-016, Lisbon, Portugal

^4^Natural History Museum of Denmark, University of Copenhagen, Copenhagen, Denmark

*Corresponding author: asreboleira@fc.ul.pt

Table S1. *Proasellus lusitanicus* (Frade, 1938) thermal rump-up experiment results. T: temperature in °C; L: body length in mm, measured from the anterior part of the head to the tip of the telson; M: dry mass in mg and OCR: oxygen consumption rates in ng O_2_/mg × h (17 °C = temperature at the collection site in the ‘Christmas siphon’ of Almonda Cave, Estremenho karst massif, Portugal). ID indicates the individuals of *P. lusitanicus* used in this study. Outliers (individuals in heat rigor) are indicated in bold.

| ID | T | L | M | OCR |
| --- | --- | --- | --- | --- |
| A2 | 17.0 | 6.2 | 9.4 | 29.5 |
| A3 | 17.0 | 7.0 | 8.7 | 51.5 |
| A4 | 17.0 | 4.8 | 4.2 | 21.3 |
| A5 | 17.0 | 6.5 | 7.4 | 46.7 |
| B1 | 17.0 | 4.5 | 1.7 | 68.7 |
| B2 | 17.0 | 5.4 | 3.3 | 188.2 |
| B3 | 17.0 | 4.7 | 1.8 | 259.5 |
| C1 | 17.0 | 4.2 | 2.4 | 104.7 |
| C2 | 17.0 | 5.1 | 9.5 | 66.3 |
| C3 | 17.0 | 6.9 | 8.8 | 46.7 |
| C4 | 17.0 | 7.2 | 9.4 | 43.9 |
| C5 | 17.0 | 6.7 | 4.8 | 167.5 |
| D1 | 17.0 | 4.2 | 2.2 | 66.8 |
| D4 | 17.0 | 4.4 | 2.2 | 40.1 |
| A2 | 19.5 | 6.2 | 9.4 | 40.7 |
| A3 | 19.5 | 7.0 | 8.7 | 23.3 |
| A4 | 19.5 | 4.8 | 4.2 | 62.6 |
| A5 | 19.5 | 6.5 | 7.4 | 48.5 |
| B1 | 19.5 | 4.5 | 1.7 | 83.8 |
| B2 | 19.5 | 5.4 | 3.3 | 80.8 |
| B3 | 19.5 | 4.7 | 1.8 | 191.4 |
| C1 | 19.5 | 4.2 | 2.4 | **0.04** |
| C2 | 19.5 | 5.1 | 9.5 | 155.9 |
| C3 | 19.5 | 6.9 | 8.8 | 43.9 |
| C4 | 19.5 | 7.2 | 9.4 | 38.1 |
| C5 | 19.5 | 6.7 | 4.8 | 42.6 |
| D1 | 19.5 | 4.2 | 2.2 | 85.2 |
| D4 | 19.5 | 4.4 | 2.2 | 86.8 |
| A2 | 21.0 | 6.2 | 9.4 | 20.7 |
| A3 | 21.0 | 7.0 | 8.7 | 35.1 |
| A4 | 21.0 | 4.8 | 4.2 | 53.7 |
| A5 | 21.0 | 6.5 | 7.4 | 21.3 |
| B1 | 21.0 | 4.5 | 1.7 | 142.4 |
| B2 | 21.0 | 5.4 | 3.3 | **0.03** |
| B3 | 21.0 | 4.7 | 1.8 | 97.3 |
| C1 | 21.0 | 4.2 | 2.4 | 88.0 |
| C2 | 21.0 | 5.1 | 9.5 | 29.2 |
| C3 | 21.0 | 6.9 | 8.8 | 63.3 |
| C4 | 21.0 | 7.2 | 9.4 | 30.3 |
| C5 | 21.0 | 6.7 | 4.8 | 63.0 |
| D1 | 21.0 | 4.2 | 2.2 | **0.01** |
| D4 | 21.0 | 4.4 | 2.2 | 107.1 |
| A2 | 22.5 | 6.2 | 9.4 | 34.3 |
| A3 | 22.5 | 7.0 | 8.7 | 26.2 |
| A4 | 22.5 | 4.8 | 4.2 | 2.9 |
| A5 | 22.5 | 6.5 | 7.4 | 23.3 |
| B1 | 22.5 | 4.5 | 1.7 | 18.2 |
| B2 | 22.5 | 5.4 | 3.3 | **0.01** |
| B3 | 22.5 | 4.7 | 1.8 | 0.1 |
| C1 | 22.5 | 4.2 | 2.4 | **0.01** |
| C2 | 22.5 | 5.1 | 9.5 | 91.9 |
| C3 | 22.5 | 6.9 | 8.8 | **0.01** |
| C4 | 22.5 | 7.2 | 9.4 | 23.3 |
| C5 | 22.5 | 6.7 | 4.8 | 56.5 |
| D1 | 22.5 | 4.2 | 2.2 | **0.01** |
| D4 | 22.5 | 4.4 | 2.2 | **0.01** |
